# CREST Calcinosis Affecting the Lumbar and Cervical Spine and the Use of Minimally-Invasive Surgery

**DOI:** 10.7759/cureus.1145

**Published:** 2017-04-08

**Authors:** Kassem Faraj, Kristin Perez-Cruet, Mick Perez-Cruet

**Affiliations:** 1 Surgery, Oakland University William Beaumont School of Medicine; 2 Neurosurgery, Oakland University William Beaumont School of Medicine; 3 Michigan Head and Spine Institute, Oakland University William Beaumont School of Medicine

**Keywords:** calcinosis cutis, minimally invasive neurosurgery, minimally invasive spine surgery, lumbar fusion, spine fusion

## Abstract

Calcinosis in CREST (calcinosis, Raynaud's phenomenon, esophageal dysmotility, sclerodactyly, and telangiectasia) syndrome can affect the spinal and paraspinal areas. We present the first case to our knowledge where a CREST syndrome patient required surgery for spinal calcinosis in both the cervical and lumbar areas. A 66-year-old female with a history of CREST syndrome presented with right-sided lower extremity radicular pain. A computed tomography (CT) scan showed bilateral lumbar masses (5.8 cm on the right, 3.8 cm on the left) that projected into the foramina and into the spinal canal. The patient underwent minimally invasive bilateral surgical resection of the paraspinal masses, posterior decompressive laminectomy, posterior interbody, and posterolateral fusion. The specimen was consistent with the calcinosis of CREST syndrome. The patient’s lumbar symptoms were relieved, however, two years later she presented with right radicular arm pain. A CT scan revealed a large lobulated benign tumor-like lesion on the left at C6-C7 encroaching upon the neural foramen and a large right lobulated lesion encroaching into the neural foramen with severe compression of the neural foramen at the C7-T1 level and extension into the canal, with anterior and posterior subluxation present throughout the cervical spine. Surgery was performed, which involved cervical mass resections, posterior spinal cord decompression, reconstruction, and fusion. The patient did well and has been symptom-free since her surgery. Calcinosis of the spine is a known entity that can cause morbidity in patients with CREST syndrome. Minimal invasive surgical approaches are effective and can be considered for some of these patients.

## Introduction

Systemic sclerosis (SSc), previously known as scleroderma, is a complex disorder with a pathophysiology that is likely autoimmune, where a combination of immune activation, vascular damage, and excess connective tissue synthesis and organ deposition leads to a wide range of symptoms [1-2]. There are two general forms of SSc: limited and diffuse [3]. The limited type, also known as CREST syndrome, includes calcinosis cutis, Raynaud’s phenomenon, esophageal dysmotility, sclerodactyly, and telangiectasia.

Calcinosis is the calcification of various tissues in the body and in SSc it classically occurs in soft tissues, commonly over the joints of the palmar hand [3]. It has been shown to be present in nearly 25% of SSc patients, being more common in the patients with the CREST type [4]. Calcinosis has been reported to occur near larger bony structures as well, such as the lumbar and cervical vertebrae, where it can result in neurological symptoms [5-8]. In these patients, surgery can be recommended, as the patient can be symptomatic and/or have extensive destruction of the vertebrae and surrounding structures [8]. Although it is well known that calcinosis can affect the various areas in the spine in CREST patients, we present the first case to our knowledge where a CREST patient required surgery for spinal calcinosis in both the cervical and lumbar areas.

## Case presentation

### History and physical

**Figure 1:**
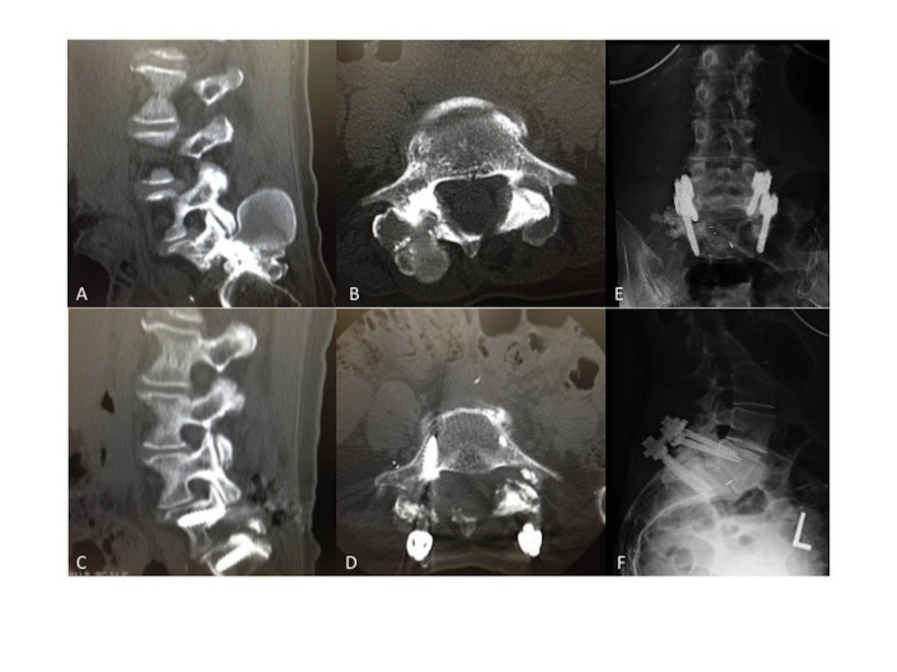
Lumbar Spine Preoperative computed tomography (CT) scan of the lumbar spine in the sagittal (A) and axial (B) section through L5-S1 illustrates two cyst-like masses associated with the L5-S1 facets that exhibit a rim-like calcification. Postoperative images illustrate successful mass resection, laminectomy and posterolateral fusion at L5-S1 in the respective planes (C, D). Post-operative sagittal (E) and coronal (F) x-rays are shown illustrating instrumentation.

A 66-year-old female with a history of CREST syndrome presented with right-sided lower back pressure with pain that radiated into the right buttock and lower extremity. On exam, the patient was tender in the lower back, with associated dysesthesia to the right side in a S1 pattern. A CT scan was performed (Figures 1A, 1B), which showed bilateral L5-S1 advanced facet arthropathy and bilateral masses (5.8 cm on the right, 3.8 cm on the left) associated with the L5-S1 facets that exhibited a rim-like calcification. The masses projected into the foramina bilaterally and into the spinal canal.

### Operation

After giving informed consent, the patient underwent minimally invasive surgery that involved bilateral surgical resection of the paraspinal masses, posterior decompressive laminectomy of L5-S1, posterior interbody fusion, and posterolateral fusion.

Using lateral fluoroscopy, the L5-S1 level was localized and a right-sided paraspinal incision was made. The fascia was entered and the cyst on the right side was completely removed in gross. The facet complex and lamina were drilled and a decompressive laminectomy was performed. Facetectomy was performed to approach the disc space and the ligamentum flavum was removed to assure adequate nerve decompression. Posterior and posterolateral fusions were then performed using the patient’s own bone and morcellized allograft. A similar approach was taken on the patient’s left side and a smaller cyst was identified and removed. Pedicle screws were then applied percutaneously using fluoroscopy.

### Postoperative care

Postoperatively, the pathology specimen was shown to consist of degenerative calcified material with macrophages and giant cells, with no cyst lining, consistent with calcinosis of the spine. The patient noted a relief of her lower extremity pain and tingling and was discharged three days later on oral pain medications. Postoperative images were eventually obtained (Figures 1C, 1D, 1E, 1F).

**Figure 2:**
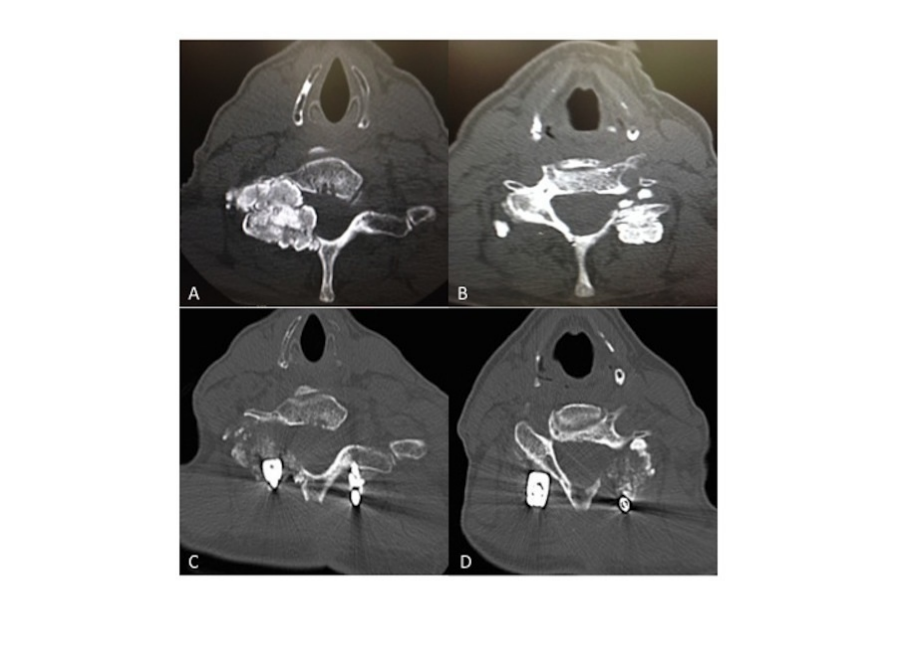
Cervical Spine Preoperative computed tomography (CT) scan of the cervical spine in two axial cuts at C6-C7 (A) and C7-T1 (B) illustrates multilobulated, calcified masses involving the facet joint on the right and left, respectively. Postoperative axial sections through C6-7 (C) and C7-T1 (D) after mass resection, spinal reconstruction and fusion with instrumentation.

Two years after this patient’s lumbar surgery, she presented with right posterior arm pain that radiated distally, with associated numbness and tingling. A CT scan (Figures 2A, 2B) revealed a large lobulated benign tumor-like lesion on the left at C6-C7 encroaching upon the neural foramen and a large right lobulated lesion encroaching into the neural foramen with severe compression of the neural foramen at the C7-T1 and extension into the canal. There was also anterior subluxation at C4-C5, C6-C7, C7-T1 and posterior subluxation at C5-C6.

After giving informed consent, the patient underwent standard open surgery, as the minimally invasive approach was not deemed appropriate due to the extensive spinal cord involvement of the masses. The procedures performed included the following: cervical mass resections, posterior spinal cord decompression, reconstruction, and fusion.

The pathology report confirmed calcinosis similar to the previous lumbar masses. The patient did well postoperatively and was instructed to follow up as an outpatient, where postoperative images were taken (Figures 2C, 2D). She is currently doing very well and has complete relief of her neurological symptoms. Imaging after nearly one year shows no evidence of recurrence.

## Discussion

We present, to our knowledge, the first reported case of CREST syndrome calcinosis affecting multiple areas of the spine in a single patient. Calcinosis of the spine is a known entity in SSc, where it is most commonly seen in the cervical and lumbar spine, and rarely in the thoracic spine [6-8].

These masses are potentially destructive to the surrounding structures. For instance, in our patient the masses in both instances were compressive on various portions of the spine and in the cervical case, there was significant projection of the mass onto the actual spinal cord, which necessitated careful microscopic resection of this area. Our patient was symptomatic with both masses, but these spinal masses can also be asymptomatic.

Calcinosis can occur in various immunological disorders, but its cause is not known. It is thought to occur in a dystrophic fashion in these disorders, where calcification is promoted in damaged or devitalized tissues in patients with normal calcium levels. In SSc, it is thought to occur in areas that experience recurrent microtrauma (i.e., fingers, forearms, elbows) [9]. With this theory, it is not surprising that calcinosis of the spine is being recognized, as the spine is a common place for microtrauma, due to the extensive flexibility and susceptibility of some areas to degenerative disc disease and intervertebral disc herniation [10-12].

Minimally invasive surgery has become a growing interest as a technique to manage spinal pathology and there are many ways that a surgeon can incorporate this technique into practice. Such uses include microdiscectomy, chemonucleolysis for disc herniation, percutaneous vertebroplasty and kyphoplasty, spinal fusion, spinal tumor resection, spinal dural arteriovenous fistula repair, and others [13]. The minimally invasive approach is widely used and has been shown to yield similar efficacy as the open approach, with less intra-operative blood loss and lower hospital lengths of stay for patients undergoing spinal fusion for spondylolisthesis [14]. The safety, efficacy, and long-term outcomes of this approach compared to conventional approaches for the various applications is currently being investigated.

Our patient was initially able to benefit from the minimally invasive approach for her lumbar masses, which allowed her to remain symptom-free until this day for the lumbosacral related symptoms. Her case illustrates how this technique can be used for various aspects (i.e., mass resection, spinal decompression, spinal fusion) in an individual operation.

## Conclusions

Calcinosis of the spine is a known entity that can cause morbidity in patients with CREST syndrome. When intervention is necessary (i.e., patients are symptomatic), surgical resection with spinal cord decompression and fusion can be effective in managing symptoms and preventing further spinal cord injury. A minimally invasive surgical approach can also be offered in patients who are candidates with less extensive spinal cord involvement.
